# Distinct impacts of feeding frequency and warming on life history traits affect population fitness in vertebrate ectotherms

**DOI:** 10.1002/ece3.10770

**Published:** 2023-11-23

**Authors:** Simon Bazin, Claire Hemmer‐Brepson, Maxime Logez, Arnaud Sentis, Martin Daufresne

**Affiliations:** ^1^ INRAE, Univ. Savoie Mont Blanc, CARRTEL Thonon‐les‐Bains France; ^2^ INRAE, Aix Marseille Univ., RECOVER Aix‐en‐Provence France; ^3^ INRAE, RIVERLY Villeurbanne Cedex France

**Keywords:** climate change, feeding frequency, integral projection model, life history traits, strategy, temperature size rule, warming

## Abstract

Body size shifts in ectotherms are mostly attributed to the Temperature Size Rule (TSR) stating that warming speeds up initial growth rate but leads to smaller size when food does not limit growth. Investigating the links between temperature, growth, and life history traits is key to understand the adaptive value of TSR, which might be context dependent. In particular, global warming can affect food quantity or quality which is another major driver of growth, fecundity, and survival. However, we have limited information on how temperature and food jointly influence life history traits in vertebrate predators and how changes in different life history traits combine to influence fitness and population demography. We investigate (1) whether TSR is maintained under different food conditions, (2) if food exacerbates or dampens the effects of temperature on growth and life history traits and (3) if food influences the adaptive value of TSR. We combine experiments on the medaka with Integral Projection Models to scale from life history traits to fitness consequences. Our results confirm that warming triggers a higher initial growth rate and a lower adult size, reduces generation time and increases mean fitness. A lower level of food exacerbates the effects of warming on growth trajectories. Although lower feeding frequency increased survival and decreased fecundity, it did not influence the effects of warming on fish development rates, fecundity, and survival. In contrast, feeding frequency influenced the adaptive value of TSR, as, under intermittent feeding, generation time decreased faster with warming and the increase in growth rate with warming was weaker compared to continuously fed fish. These results are of importance in the context of global warming as resources are expected to change with increasing temperatures but, surprisingly, our results suggest that feeding frequency have a lower impact on fitness at high temperature.

## Graphical abstract

We investigated under laboratory conditions the independent and interactive effects of temperature and feeding frequency on the growth, fecundity, and survival of the medaka fish *Oryzias latipes*. We next used our empirical estimates of vital rates as input parameters of an Integral Projection model (IPM) to predict how modifications in vital rates translate into population demographic parameters.

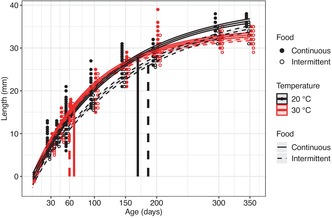

## 1 INTRODUCTION

**FIGURE 1:**
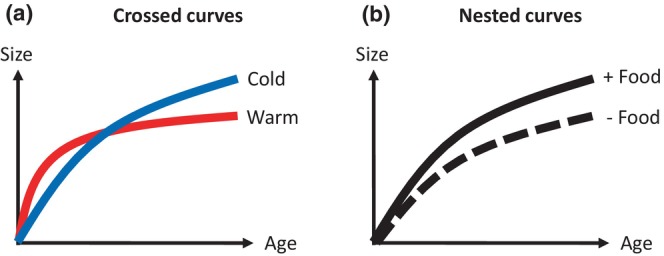
Patterns of crossed vs. nested growth curves driven by (a) temperature and (b) feeding frequency (after Berrigan & Charnov, 1994).

Body size reduction has been proposed as a third universal species response to global warming (Daufresne et al., 2009; Gardner et al., 2011; Sheridan & Bickford, 2011), in addition to changes in phenology (Visser & Both, 2005) and geographic distribution (Parmesan & Yohe, 2003). While the first two responses have been studied extensively (Meyer et al., 1999), the third one has received less attention despite its high prevalence and magnitude. For instance, body size can reduce up to −4% per °C in terrestrial species and up to −8% per °C in aquatic ectotherms (Forster et al., 2012). Previous studies focused on proximal mechanisms, (i.e., how environmental factors influence life history traits by impacting physiological and developmental processes [Thierry, 2005]) and ultimate mechanisms related to the evolution and adaptive value of body size changes (Atkinson & Sibly, 1997; Frazier et al., 2001; Hoefnagel & Verberk, 2015; Verberk et al., 2021; Walczyńska et al., 2015; Zuo et al., 2012) and their variability among species and habitats (Atkinson, 1994; Forster et al., 2012; Horne et al., 2015). At the individual level, body size shift can be explained by the Temperature Size Rule (TSR, Angilletta et al., 2004; Arendt, 2007, 2011; Atkinson, 1994; Atkinson & Sibly, 1997; Berrigan & Charnov, 1994; Perrin, 1995), which states that ectotherms grow faster but reach a smaller size at a given stage of development (e.g. size at maturity or adult size) under warm environment compared with colder ones, resulting in “crossed” growth curves (Figure 1). This pattern of TSR remains an evolutionary puzzle (Atkinson & Sibly, 1997) and body size shifts could be the result of different developmental strategies. For example, a recent study showed that warming accelerates growth and reproduction leading to a rapid life cycle but also a decrease in adult survival in a temperate lizard species (Bestion et al., 2015). This study and others (Clissold & Simpson, 2015; Corrêa et al., 2021; Courtney Jones et al., 2015; Kingsolver et al., 2006; Marn et al., 2017; Rohner et al., 2017) suggest that it is important to investigate the links between growth trajectories and fitness related traits (survival and fecundity) to better understand how the combination of these traits may influence individual fitness and population demographic parameters. However, most studies on TSR did not investigate these links (but see Corrêa et al., 2021; Kingsolver et al., 2006; Marn et al., 2017) and the evolutionary puzzle of the TSR thus remains open. We argue that investigating the links between temperature, growth, and life history traits is key to understand the adaptive value of TSR. In addition, the adaptive value of TSR might be context dependent which calls for investigating (1) how other stressors interact with temperature, (2) if they can amplify or dampen thermal effects, and (3) if this can change the adaptive value of the TSR.

Besides temperature, another major factor underlying growth, reproduction, and survival is food (Boersma & Vijverberg, 1996; Boggs & Ross, 1993; Corrêa et al., 2021; Giberson & Rosenberg, 1992). Individuals need enough resources, as energy and material inputs, to sustain their metabolic demand and optimize the allocation of energy to growth, reproduction, and maintenance (Brown et al., 2004; Cross et al., 2015; Lemoine & Burkepile, 2012). There is a long history of research on the influence of food on the growth rate and fecundity of ectothermic species (Boersma & Vijverberg, 1996; Corrêa et al., 2021; Giberson & Rosenberg, 1992; Johnston et al., 2002; Rasmussen & Ostenfeld, 2000). In most cases, individuals with a higher food level have a higher fecundity and have both a higher initial growth rate and a larger size at age compared to individuals under limited food conditions. In contrast to the pattern of crossed curves driven by temperature, different food levels lead to a pattern of nested curves where the growth curve under limiting food is nested below the growth curve under unlimited food (Figure 1). Interestingly, food restriction may also be beneficial to the life span of organisms as this restriction reduces the production of senescence‐accelerating oxidizing agents during metabolism (Gredilla et al., 2001; Sohal & Weindruch, 1996; Speakman, 2005), resulting in a “eat little die old” strategy. The effects of food on fecundity (which decreases) and survival (which increases) are thus opposite and can be explained by a resource distribution to nutrient‐limited processes (Corrêa et al., 2021). As for temperature, this indicates that we should consider the effects of food on multiple life history traits to better identify fitness consequences and thus evolutionary strategies. Because food is a key universal requirement of species niche, investigating the effects of food and temperature on life history traits is a natural step for our understanding of the factor that can modulate the adaptive value of TSR.

Temperature and food availability are key drivers of species geographical distribution, primary production, species interactions, and energy and material fluxes (Elser et al., 2007; Enquist et al., 1999; Falkowski et al., 1998; Thomas et al., 2017) and are at the core of influential ecological theories. On one side, temperature is fundamental to the metabolic scaling theory (Brown et al., 2004) while, on the other side, food is at the core of resource competition theory (Tilman, 1982) and ecological stoichiometry theory (Sterner & Elser, 2017). Better understanding the interactions between temperature and food is thus crucial for anticipating ecological responses to multiple drivers of global change, such as climate warming and elevated nutrient supply. The interactive effects of temperature and food on life history traits have been studied in many organisms such as phytoplankton (Huey & Kingsolver, 2019), diatoms (Thomas et al., 2017; Walczyńska & Sobczyk, 2017), rotifers (Kiełbasa et al., 2014), cladocerans (Betini et al., 2020; Giebelhausen & Lampert, 2001; Persson et al., 2011; Wojewodzic et al., 2011), aquatic insect larvae (Giberson & Rosenberg, 1992), terrestrial insects (Clissold & Simpson, 2015; Corrêa et al., 2021; Kingsolver et al., 2006; Kingsolver & Woods, 1998; Lee & Roh, 2010; Petersen et al., 2000; Rohner et al., 2017), fish (Brett, 1971; Brett et al., 1969; McLeod et al., 2013), and turtles (Marn et al., 2017). In these studies, warming generally resulted in a rapid life cycle by increasing growth rates and decreasing age and size at maturity as well as survival probabilities. However, these thermal effects were often modulated by food level. In particular, temperature and food can covary and impact ectotherm life history traits. Koussoroplis and Wacker (2016) showed that the effect of food on life history traits is more severe when temperature moves away from the optimal temperature. Nevertheless, these previous studies did not fully investigate how the effects of temperature and food on multiple life‐history traits combine to influence fitness and population demographic parameters (e.g., generation time and population growth rate). This is an important limitation as we need to determine how the combination of effects on multiple traits influence fitness to understand the adaptive value of plastic and evolutionary responses to environmental factors; the latter being the focus of several studies and intense debates in the literature on TSR (see Fryxell et al., 2020; Kingsolver & Huey, 2008; Walters & Hassall, 2006; Zamudio et al., 1995). In addition, many of the studies mentioned above were conducted on small invertebrate species (but see Brett, 1971; Brett et al., 1969; Marn et al., 2017; McLeod et al., 2013). As a result, we have limited information on how temperature and food jointly influence life history traits of vertebrate predators. This is of importance as we expect body size changes to be stronger in predatory species (Forster et al., 2012), which could affect population structure, trophic interaction strength, and food webs stability (Emmerson & Raffaelli, 2004; Osmond et al., 2017; Sentis et al., 2017; Uszko et al., 2022).

In this study, our objectives were to investigate (1) whether TSR is maintained under different food conditions, (2) if food exacerbates or dampens the effects of temperature on the growth trajectory and life history traits of a vertebrate predatory species, and (3) if food can influence the adaptive value of TSR. We address these three points by combining experiments on the medaka fish (*Oryzias latipes*, Temminck & schlegel) with Integral Projection Models (IPMs) to scale from life history traits to fitness consequences. In particular, we first experimentally investigated the growth, reproduction, and survival probability of medaka fish raised at two temperatures (20°C and 30°C) and fed at two feeding frequencies (continuous or intermittent). We next used our empirical estimates to parameterise an IPM that was then used to understand and predict how temperature and feeding frequency combine to influence mean fitness and generation time. We hypothesized that warming would increase growth and fecundity but lower survival, leading to a rapid life cycle (short generation time). Moreover, we hypothesized that this thermal effect would be modulated by intermittent feeding, the latter would increase survival and selects for late maturation at larger body size. We therefore expected that, in contrast to temperature, intermittent feeding would increase the population generation time. As a result, temperature and intermittent feeding would have opposite effects on generation time and their overall effect would thus depend on the relative strength of their individual effects. Overall, our aim was to better understand to which extent investigating growth, reproduction, and survival patterns could help disentangling the relative impacts of temperature and food on body size shifts under global warming as well as understand the adaptive values of these phenotypic responses.

## 2 MATERIALS AND METHODS

### 2.1 Biological system and rearing conditions

The medaka is a small iteroparous freshwater fish native to East Asia (Hirshfield, 1980). The life span of a medaka is about 2 years and its adult size varies between 30 and 50 mm (Ding et al., 2010; Egami & Etoh, 1969). This is an eurythermal species living in small ponds and rice paddies with temperatures ranging from 10°C to 34°C (Terao, 1985), with an optimum individual growth temperature of 25°C (Dhillon & Fox, 2004; Hirshfield, 1980). At this temperature, the medaka requires only 10–12 weeks to reach sexual maturity. Fish were maintained in the laboratory in 20 L aquaria with 20–30 fish using an open water system with a water supply controlled by drip emitters (1 L h^−1^). This density (less than 2–3 fish L^−1^) does not cause any stress or agonistic behavior in this species (Denny et al., 1991). Input water had a pH of 7.5 at 16 °GH and its quality was maintained with mechanical, biological, and UV filtration. Each tank (25 × 40 × 20 cm) was equipped with an air filter to prevent high nitrite concentrations and maintain oxygen at saturation.

The parental F_0_ generation consisted of 46 females and 30 males (approximately 120 days old) belonging to the CAB strain from Carolina Biological Supply Company (Burlington, NC, USA) and provided by AMAGEN© (Gif‐sur‐Yvette, France). At AMAGEN©, the fish are fed ad libitum, providing a portion of ~1% of their weight four times a day. At reception, fish were kept for 5 days at 25°C. Then, half of the fish were placed into five 20 L tanks for the “cold” thermal regime and the other half were placed into five 20 L tanks for the “warm” thermal regime. The female‐to‐male sex ratio per tank ranged from 1.33:1 to 1.66:1. The tank temperatures were increased or decreased by 0.5°C every day until they reached 30°C or 20°C. During this acclimation period, the photoperiod was 12 h:12 h (day:night) and, after acclimation, it was then adjusted to 16 h:8 h (day:night) which is optimal for medaka reproduction (Hirshfield, 1980).

From this F_0_ generation, about 300 eggs were collected in each tank and placed into hatcheries. Egg collection was carried out over 5 days, implying that the maximum age difference between two fish was 5 days. Once the eggs had hatched, F_1_ fish larvae were transferred into six small nurseries (2.5 L) per rearing temperature. After 30 days of growth, the fish larvae were transferred from their nursery to a 20 L tank and reared under four different treatments: conti_20 (continuous and 20°C), inter_20 (intermittent and 20°C), conti_30 (ad libitum and 30°C), and inter_30 (intermittent and 30°C). Thus, for each treatment, approximately 80 fish were maintained in three tanks, and their growth was monitored, except for inter_20 where only 54 fish in two tanks could be kept. The fish were fed with TetraMin© every morning (for the continuously fed fish) or every two mornings (for the intermittently fed fish). On each feeding day, TetraMin© was provided to each tank until the fish no longer went up to the surface to get food. Excess food was systematically removed after feeding to prevent feeding between two meals. Apart from temperature and food, all the experimental parameters were similar in the four treatments.

The species‐specific optimal thermal range for TSR is the range between the temperature at which the population growth rate becomes positive and the temperature at which the population growth rate is maximal (Walczyńska et al., 2016). Walczyńska et al. (2016) showed that TSR can be maintained for temperatures slightly above the optimal temperature for population growth rate. The optimal temperature for medaka reproduction is 27°C (Hirshfield, 1980; Yamamoto, 1975), and our warm experimental temperature is only 3°C above this optimal temperature which suggests that our warm temperature is within the “optimal thermal range” for TSR and that our results are not the product of a response to a thermal stress.

In the next sections, we explain how the effects of temperature and food on growth, fecundity, and survival were measured and analyzed and how we used these empirical estimates to parameterize an IPM model to understand how effects on growth and life history traits combine to influence mean fitness and generation time.

### 2.2 Growth, fecundity, and survival

The total length (from the head to the tip of the caudal fin, TL) of fish was measured with a precision of 0.5 mm at 30, 45, 60, 100, 150, 200, 300, and 350 days post hatching. Fish were measured after placing them on a 5 cm diameter Petri dish layered with a millimeter graph paper and filled with water. They were then immediately released into their respective tank. We measured between 12 and 17 fish per tank at a given age or all the fish when the number of survivors was lower (see Figure S1 for more details). As fish were not identified individually, the growth curves apply to the experimental population (i.e., one curve per treatment) and not to individuals. In each tank, we determined the time at which the first individual fish was sexually mature, and we measured its size and determined its age. The investment in reproduction was quantified by counting each day in each tank the number of females with eggs and the number of eggs laid per female (all the eggs were systematically removed). The survival probability from 60 days (age of the first individual sexually mature all treatments combined), referred to as survival in this study, was monitored daily until the end of the experiment.

### 2.3 Statistical analysis

TL measurements and ages were used to fit Von Bertalanffy growth curve model (Von Bertalanffy, 1938):

(1) $$L_{t}=L_{\infty }\left(1-e^{-K\left(t-t_{0}\right)}\right)$$

where *L*_*t*_ is the estimated total length at time *t*, *L*_∞_ the maximum asymptotic size (i.e., the total length for fish with an ∞ age), *K* is the initial growth rate, and *t*_0_ is the theoretical age at which body size is null.

Von Bertallanfy growth curves parameters (*L*_*∞*_, *K*, *t*_0_) were estimated by Bayesian inference using the Bayesian software JAGS and the “R2jags” package (Su & Yajima, 2015) in R software (version 4.0.2; R development Core Team). We assumed that the asymptotic size *L*_*∞*_, the initial growth rate *K*, and the theoretical age at null size *t*_0_ could vary between temperature (*T*) and food (*C*) conditions. Consequently, four values of *L*_*∞*_, *K*, and *t*_0_ (one for each combination [*CT*] of temperature and food condition) were fitted. For each parameter, we used a normal uninformative prior with a mean of 0 and a precision parameter (inverse of the variance) of 0.001:

(2) $$L_{\infty \mathrm{CT}}N\left(0,0.001\right)\;K_{\mathrm{CT}}N\left(0,0.001\right)\;t_{0\mathrm{CT}}N\left(0,0.001\right)$$

To account for tanks (*t*) variability, we estimated random effects *ε* for each parameter using a multivariate normal distribution, *ε* ~ N(0, *∑*). The covariance matrix *∑*_(3,3)_ was defined as:

(3) $$\left|\begin{array}{ccc} \sigma_{L_{\infty }}^{2} & r_{1}\cdot \sigma_{L_{\infty }}\cdot \sigma_{K} & r_{2}\cdot \sigma_{L_{\infty }}\cdot \sigma_{t_{0}}\\ r_{1}\cdot \sigma_{L_{\infty }}\cdot \sigma_{K} & \sigma_{K}^{2} & r_{3}\cdot \sigma_{K}\cdot \sigma_{t_{0}}\\ r_{2}\cdot \sigma_{L_{\infty }}\cdot \sigma_{t_{0}} & r_{3}\cdot \sigma_{K}\cdot \sigma_{t_{0}} & \sigma_{t_{0}}^{2} \end{array}\right|$$

With $\sigma_{L_{\infty }}$, $\sigma_{K}$, and $\sigma_{t_{0}}$ the standard deviations of each random vector, one per parameter, and *r*_1_, *r*_2_, *r*_3_ the correlations between these vectors. We used uninformative priors with a uniform distribution for each parameter of *∑*, adapting the limits to the parameters (e.g., between −1 and 1 for a correlation).

$L_{\infty \mathrm{CT}}$, $K_{\mathrm{CT}},$ and $t_{0\mathrm{CT}}$ are thus hyperpriors (population parameters) that serve to assess parameters for each tank (*t*) when associated with the random effects. For instance for the $L_{\infty }$ parameter:

(4) $$L_{\infty t}=L_{\infty \mathrm{CT}}+\varepsilon_{L_{\infty }t}$$

We then used (Equation 1) to estimate the expected mean total length $L_{\mathrm{tj}}$ for each tank (*t*), and each age (*j*):

(5) $$L_{\mathrm{tj}}=L_{\infty t}\left(1-e^{-K_{t}\left(t_{j}-t_{0t}\right)}\right)$$

Finally, we hypothesized that the observed total length of each fish (*f*), *L*, was normally distributed:

(6) $$\begin{array}{c} L_{\mathrm{ftj}}N\left(L_{\mathrm{tj}},\sigma \right)\\ \mathrm{\sigma U}\left(0,10\right) \end{array}$$

To compare the growth patterns among temperature and food conditions, we plotted the average growth curves for each treatment (combination of food condition and temperature) and their credibility interval (CI) using the posterior distributions of the parameters (*L*_*∞CT*_, *K*_*CT*_, *t*_0*CT*_) that were obtained from five independent Monte‐Carlo Markov chains (see Figure S2 for more details on the estimated parameter values). For each chain, after an initial burning of 50,000 values, 400,000 iterations were computed and we conserved one value every 200 iterations to limit autocorrelation between estimations. The posterior distributions for each average total length at age (*L*) were thus constituted of 10,000 values. The quantiles 2.5% and 97.5% were used to estimate credibility intervals CIs. We compared the growth curves among our four experimental treatments by investigating the overlap among their CIs. Curves were considered significantly different when their CIs did not overlap (Pritchard et al., 2017).

We investigated the effects of temperature, feeding frequency, and their interaction (fixed effects) on mean daily clutch size per female (log‐transformed) and survival probabilities using a linear mixed effects model (*lmer* function in the “lme4” package, Bates et al., 2015) and a mixed effects Cox proportional hazards model (*coxme* function in the “coxme” package [Therneau, 2015]), respectively, with tank as a random factor. For both models, analyses of deviance using Wald tests were provided to test the significance of fixed parameters. We tested the assumptions of the mixed effects Cox proportional hazards model using the *cox.zph* function (“survival” package [Therneau & Lumley, 2015]) which correlates the corresponding set of scaled Schoenfeld residuals with time to test for independence between residuals and time (see Figure S3 for more details).

### 2.4 Integral projection modelling (IPM)

Integral Projection Models are discrete‐time, structured population models that estimate the asymptotic behavior of populations by combining life history traits that can be discrete or continuous (Levin et al., 2021). We used our empirical measurement of life history traits to quantify the fitness of populations simulated by IPMs for our four experimental treatments of temperature and food. To build an IPM, the first step is to represent the life cycle of the focal species. At each time step, an individual medaka has a probability *s* to survive. If it survives, it grows according to a growth function *g*. This individual has a chance to reproduce according to the function *f_p*, and if it reproduces, it produces a number of eggs according to the fecundity function *f_n*. In the model, the vital rates (*s*, *g*, *f_p*, *f_n*) are functions of the fish body size at time *t*. The eggs have hatching and survival probabilities according to the function *f_g*, and the resulting juvenile fish have a size distribution *f_d*. Egg hatching rate, survival of juvenile, and their size distribution are independent of the size of their parents.

We used a similar IPM structure as in Bogdan et al. (2021):

(7) $$n\left(z^{\prime },t+1\right)=\int_{L}^{U}K\left(z^{\prime },z\right)n\left(z,t\right)\mathrm{dz}$$

where *n*(*z*′, *t* + 1) is the size of the population at time *t* + 1, *z*′ is the state variable describing the population (i.e., body size in our model). *n*(*z*′, *t + 1*) is obtained by integrating the product of *K*(*z*′,*z*) and *n*(*z*,*t*) over the domain [*L*, *U*]. In our model, the lower bound *L* is the minimum fish size and the upper bound *U* is the maximum size. *K*(*z*′,*z*) is a bivariate kernel function that describes the transitions to state *z*′ given the initial state of an individual *z* at time *t*. *K*(*z*′,*z*) consists of two sub‐kernels *P* and *F*. *P* describes the survival and growth of fish at time *t* (*P* = *s* ∙ *g*) and *F* describes the number and body size of juveniles at time *t* + 1 according to reproduction probability, hatching rate, juvenile survival and body size distribution (*F* = *f_p* ∙ *f_n* ∙ *f_g* ∙ *f_d*).

This yields to:

(8) $$n\left(z^{\prime },t+1\right)=\int_{L}^{U}\left[P\left(z^{\prime },z\right)+F\left(z^{\prime },z\right)\right]n\left(z,t\right)\mathrm{dz}$$

The analytical solutions of IPMs are very resource expensive. An alternative method to solve Equation 8 is to use the integration rule of the midpoint of the meshes along the domain [*L*,*U*] (Ellner et al., 2016). In our model, the domain extends from the predicted size in log of a fish after 30 days (*L*) to the maximum observed size in log (*U*). The number of meshes along this domain was set to 400.

To obtain the survival function *s*, we used Kaplan–Meier estimates to compute the survival probability for each sampled age. We then associated survival probabilities to fish body size using the estimated age–size relationship from the fitted Von Bertalanffy model. Survival probability (*s*) in function of body size was estimated using a logistic equation for each experimental treatment of temperature and feeding frequency.

To obtain the growth function *g*, we predicted the size at *t* (*L*_*t*_) (from 0 to 350 days) of 10,000 fish from the 10,000 combinations of Von Bertallanfy parameters from the Bayesian model posterior distributions. We then calculated the size at *t* + 1 (*L*_*t*+1_) from *L*_*t*_ following the formula:

(9) $$L_{t+1}=L_{t}e^{-K}+L_{\infty }\left(1-e^{-K}\right)$$

For each age, we computed the standard deviation of the sizes at *t* + 1 (10,000 values) and then considered the average value of the standard deviations to implement residual variation around growth (*g*).

For the reproduction probability (*f_p*), we used a logistic equation considering that all fish reproduce once they reach their treatment‐dependent age at maturity. For the fecundity function (*f_n*), we used a Poisson regression model to describe the link between fish size and egg number. Egg hatching rate and survival probability (*f_g*) and the body size distribution of juveniles (*f_d*) were estimated from unpublished data from the same experimental populations.

We used the “ipmr” R package (Levin et al., 2021) functions to define the kernels (*define_kernel*), the domain (*define_domains*), and the initial state of the population (*define_pop_state*) and to compute the IPMs (*make_ipm*). The number of iterations of the IPMs was fixed per treatment to achieve asymptotic dynamics according to the *is_conv_to_asymptotic* function. We used the *gen_time* and *lambda* functions from the “Rage” and “ipmr” R packages (Jones et al., 2022; Levin et al., 2021) to quantify the generation time *T* and the asymptotic *per capita* population growth rate *λ*. We quantified the uncertainty of *T* and *λ* by bootstrapping 1000 combinations of *L*_*∞*_, *K*, and *t*_0_ from the Bayesian model posterior distributions (with replacement) and by using 1000 random sample of each vital rate data set (survival, reproduction, and fecundity) and refitting all demographic functions *s*, *g*, *f_p*, *f_n*. For each new iteration, we ran an IPM and estimated *T* and *λ*. This yielded 1000 estimates of *T* and *λ* for each experimental treatment. We next calculated the 95% confidence intervals of *T* and *λ* and compared their mean values across experimental treatments based on the overlap of their 95% confidence intervals. We also performed a sensitivity analysis to investigate the sensitivity of *T* and *λ* to small changes in the vital rate estimates (see Figure S5). Data and scripts used to build the IPMs and perform the sensitivity analysis are available online.

## 3 RESULTS

**FIGURE 2:**
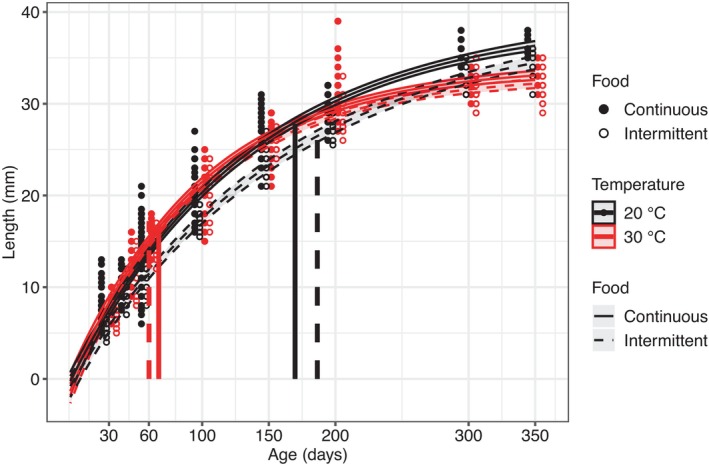
Fitted Von Bertalanffy growth curve for each combination of temperature and feeding conditions. Black and red colors represent the cold and warm treatments (i.e., 20°C and 30°C), respectively. Solid and dotted lines represent the continuous and intermittent feeding treatments, respectively. Vertical bars represent the age of the first sexually mature fish in each treatment. As fish were not identified individually, jittered points represent experimentally measured sizes (in mm) at age (in days) of fish from different replicates (i.e., tanks).

We found that when fish were fed continuously, warming leads to crossed growth curves by increasing the initial growth rate and decreasing adult size (Figure 2). The same pattern was observed under intermittent feeding, although the curves crossed later for the intermittently fed fish compared with continuously fed fish. Intermittent feeding in the cold treatment leads to nested growth curves throughout the experiment by decreasing the initial growth rate and adult size. Growth curves also tended to be nested in the warm treatment although the credibility intervals overlapped until day 149 and the curves were only significantly different toward the end of the experiment (from day 149 to day 316, Figure 2).

**FIGURE 3:**
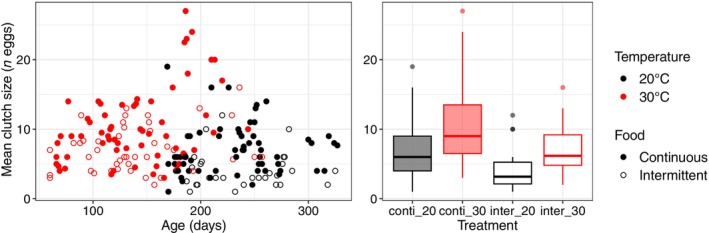
Temperature and feeding frequency effects on mean daily clutch size per female. Black and red colors represent the cold and warm treatments (i.e., 20°C and 30°C), respectively. Filled and empty points and boxplot represent the continuous and intermittent feeding treatments, respectively.

In the warm treatment, the first sexually mature continuously fed fish were observed at 67.3 ± 2.3 days (body length: 16.8 ± 0.1 mm) and at 60 days for all replicates (body length: 17.2 ± 0.7 mm) under intermittent feeding. In the cold treatment, they were sexually mature at 169.7 ± 0.6 days (body length: 26.3 ± 0.6 mm) and 186.5 ± 0.7 days (body length: 25.7 ± 0.4 mm) under continuous feeding or intermittent feeding conditions, respectively. We found that the mean daily clutch size per female was higher in the warm treatment (Figure 3; χ^2^ = 13.3, df = 1, *p* < .001, *n* = 196) and lower under intermittent feeding (χ^*2*^ = 10.6, df = 1, *p* = .001, *n* = 196). The mean daily clutch size per female was not dependent on the interaction between temperature and feeding frequency (χ^2^ = 0.8, df = 1, *p* = .374, *n* = 196).

**FIGURE 4:**
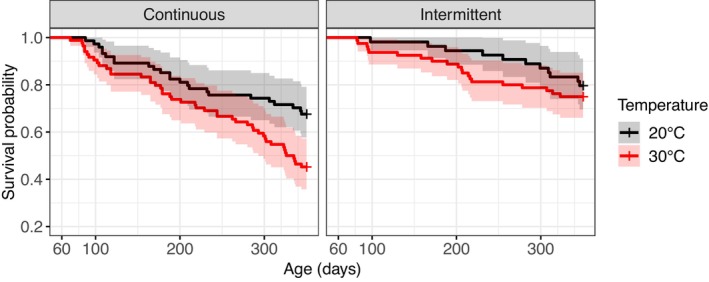
Kaplan–Meier survival curves from 60 days for each combination of temperature and feeding frequency. All fish (males and females) were included. Black and red colors represent the cold and warm treatments (i.e., 20°C and 30°C), respectively. Solid and dashed lines represent the continuous and intermittent feeding treatments, respectively. Shaded areas around the lines represent the 95% confidence intervals.

The fish survival was not significantly affected by the interaction between warming and feeding frequency (χ^2^ = 0.7, df = 1, *p* = .402, *n* = 292). In contrast, warming significantly reduced fish survival (Figure 4; χ^2^ = 7.0, df = 1, *p* = .008, *n* = 292). Moreover, intermittent feeding significantly increased survival (χ^2^ = 15.0, df = 1, *p* < .001, *n* = 292).

**FIGURE 5:**
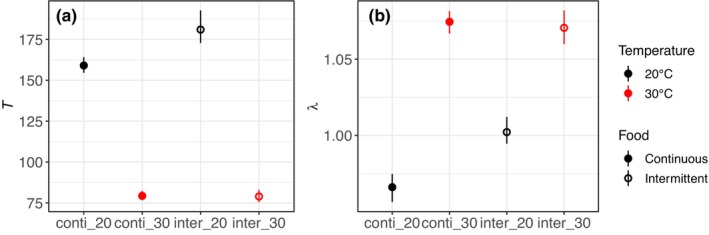
Estimated (a) generation time *T* and (b) asymptotic *per capita* population growth rate *λ* for each combination of temperature and feeding frequency. Black and red colors correspond to the cold and warm treatments, respectively. Filled and empty circles correspond to the median of continuous and intermittent feeding treatments, respectively. Bars represent 95% confidence intervals.

We found that warming decreased generation time *T* and increased the asymptotic *per capita* population growth rate *λ* (Figure 5). In the cold treatment, intermittent feeding significantly increased *T* and *λ*. Intermittent feeding had no significant effect on *T* and *λ* in the warm treatment as their 95% confidence intervals overlapped.

## 4 DISCUSSION

The results of our laboratory experiment indicate that, in agreement with the TSR rule (Arendt, 2011; Atkinson & Sibly, 1997; Berrigan & Charnov, 1994), warming leads to crossed growth curves with individuals growing faster but reaching a smaller size at maturity and adult size compared with the cold condition. In addition, we found this pattern in both feeding treatments, which indicates that TSR can be maintained under different food conditions. We also found that intermittent feeding does not affect size at maturity but leads to nested curves where intermittently fed fish are smaller than continuously fed fish at each age. Furthermore, the effects of intermittent feeding appeared to be greater at 20°C where the growth curve for the intermittent fed fish was more nested (i.e., below the curves for continuously fed fish) than at 30°C. This is surprising because we expected intermittent feeding to have more effect in warm treatment (as reported in Giberson & Rosenberg, 1992; McLeod et al., 2013; Persson et al., 2011; Wojewodzic et al., 2011) because warming increases metabolic rates which implies higher energetic demand and feeding rate to sustain high metabolic costs (Brown et al., 2004). For instance, Betini et al. (2020) found a TSR amplification in *Daphnia magna* under low food levels with a body size reduction under warming five times stronger under food restriction than under unlimited food conditions. Brett et al. (1969) found that food limitation more strongly reduced growth rates in sockeye salmon (*Oncorhynchus nerka*) at intermediate temperatures, and that optimal growth temperature shifted to lower values when food was limited. The important variability in the responses of size at maturity or adult size to temperature and food shows the complexity of understanding the mechanisms underlying the control of body size in ectotherms. We conducted a short synthesis of the results of previous experimental studies investigating the responses in size at maturity or adult size to warming and food conditions (see Table S1). These results suggest that temperature‐induced body size shifts depend on the frequency (McLeod et al., 2013), the quantity (Brett, 1971; Brett et al., 1969; Courtney Jones et al., 2015; Kingsolver & Woods, 1998) but also the quality (Kiełbasa et al., 2014; Persson et al., 2011; Wojewodzic et al., 2011) of resources, with lower resource quality amplifying the detrimental effect of warming as reported in a recent study (Sentis et al., 2022). Overall, decreasing feeding frequency tended to exacerbate the effects of warming on growth trajectories, which would indicate that we underestimate the potential of global warming for decreasing body sizes when we consider unlimited food.

We found that fish reared at 30°C were sexually mature at a younger age and produced a larger mean daily clutch size per female. These results are in line with Betini et al. (2020), Giebelhausen and Lampert (2001), and Marn et al. (2017) who found early maturation and increased fertility under warming in *Daphnia* and turtles. In contrast, less is known about the responses of developmental rates and fecundity to covariation between temperature and food. Our results suggest that developmental rates and fecundity are not sensitive to covariation between warming and feeding frequency as we did not find any effect of intermittent feeding on age at maturity and that intermittent feeding decreased mean daily clutch size similarly in both temperature conditions. We hypothesized that this last result may be explained by a temperature‐independent decrease in the energy allocated to reproduction in response to lower feeding frequency. Our results contrast with Betini et al. (2020) who found later maturation and lower fecundity in *Daphnia* under food restriction, and these detrimental effects of food were exacerbated at high temperatures. Marn et al. (2017) found a synergistic effect of the two environmental factors on reproduction with similar predictions of reproductive output under low temperature and high food availability versus high temperature and low food availability. The various responses of development rates and fecundity to covariation between temperature and food may be explained by a difference in the energy allocation to growth or reproduction under different environmental conditions, requiring further studies to fully understand them.

In our experiment, the survival probability was influenced by both temperature and feeding conditions. Indeed, fish reared at 30°C had a lower survival than fish reared at 20°C while intermittent feeding increased the survival under both temperature conditions. This beneficial effect of lower food conditions on survival was also observed in frog larvae (Courtney Jones et al., 2015) and *Daphnia* (Betini et al., 2020). Lower food implies a decrease in metabolism and thus a lower production of oxidizing agents which contributes to slow down senescence and increase survival, resulting in a “eat little die old” strategy (Gredilla et al., 2001; Pifferi et al., 2018; Sohal & Weindruch, 1996; Speakman, 2005). Although survival probability was not significantly affected by the interaction between warming and feeding frequency (i.e., the survival probability of continuously fed fish at 20°C was similar to that of fish intermittently fed fish at 30°C), our results potentially illustrate different developmental strategies. For example, at 30°C, fish may have maintained a high growth rate despite intermittent feeding in order to maintain a rapid life cycle, at the expense of lower survival. This hypothesis is supported by the fact that mortality was higher and sexual maturity was reached at a younger age and smaller size at 30°C compared with 20°C. Ultimately, measuring the fitness of the fish under the different conditions would help in understanding if these strategies are adaptive or result from physiological constraints that are difficulty overpassed by evolutionary adaptations.

When we integrated our experimentally measured traits into Integral Projection Model, we found a reduction in generation time and an increase in the population growth rate under warming, suggesting that TSR increases the adaptive value of the population. Although survival probability was lower under warming, fish reached sexual maturity much faster and had higher fecundity. The earlier sexual maturity of fish enabled them to reproduce for a longer time (Cole, 1954). Therefore, each female could produce a higher number of juveniles, which leads to a higher population growth rate compared to cold‐acclimated populations. Consistent with our experimental data, intermittent feeding increased generation time and asymptotic *per capita* population growth rate at 20°C, whereas it had no significant effect on demographic parameters of the populations at 30°C. Our experimental results showed that intermittent feeding slightly decreased fecundity but strongly increased fish survival probability, resulting in longer individual lifespans and the production of more juveniles. Ultimately, intermittent feeding proved evolutionarily advantageous in the cold treatment, leading to a population growth rate equal to unity (*λ* = 1). This indicates that the population moves from a declining dynamic (*λ* < 1) when food is not limiting to an increasing dynamic (*λ* > 1) under lower food conditions. Consequently, feeding frequency influenced the adaptive value of TSR, as, under intermittent feeding, generation time decreased faster with warming and the increase in growth rate with warming was weaker compared to continuously fed fish. Our sensitivity analyses revealed that the demographic parameters were mainly sensitive to the reproduction and survival probabilities (see Figure S5). These parameters determine the lifespan of the fish and the duration of their reproduction. The high sensitivity of the model to the reproduction probability can be explained by our assumption that, in the model, all females reproduce after reaching maturity (because we lacked information on which female reproduces when) which lead to a steep reproduction function. Nevertheless, this assumption was similar for the four treatments and should not influence the qualitative comparison of our four treatments. We suggest that the increase in population growth rate and decrease in generation time at 30°C represent a TSR‐induced increase in population adaptive value, but we were not able to disentangle the effects of warming on growth from its effects on other life‐history traits, which may affect demographic parameters. Thunell et al. (2023) and Smallegange et al. (2017) parameterized a DEB‐IPM model, incorporating parameters related to metabolism, energy allocation, and consumption to explore the consequences of temperature and food on population demographic parameters. This type of model could better distinguish the effects of TSR from other temperature effects on life history traits and investigate their relative contributions in estimating population demographic parameters. However, this type of model requires a very large number of parameters associated with each process (metabolism, energy allocation, and consumption), and we had no such information from our empirical data. Moreover, these parameters can be affected by temperature acclimation, as shown in Sentis et al. (2015), Gray (2013), and Sohlström et al. (2021). Consequently, we decided instead to use only our empirical data to parameterize our simpler IPM model, rather than making numerous, potentially less robust assumptions about the parameters required for DEB‐IPM. Although our IPM model is simpler than the DEB‐IPM model, it still allows us to explore how empirically studied life‐history traits may affect population demographics. As population growth rate corresponds to the population mean fitness, the IPM helps determining the adaptive value of TSR at different feeding frequency which is key to understand if TSR is an ultimate or proximal mechanism.

Although intermittent feeding strongly affected survival, its effects on growth, fecundity, and demographic parameters were weak, especially at 30°C. This may be explained by the potential acclimation of medaka to rearing temperatures or by feeding frequency being not severe enough. Reducing feeding events by half (1 out of 2 mornings) was considered restrictive although we cannot exclude compensatory mechanisms where intermittent‐fed fish would feed more when they have access to food (Kingsolver & Woods, 1998). In addition, temperature and feeding frequency are likely to have independent and interactive nonlinear effects on life history traits, as reported by Brett (1971). However, our experimental 2 × 2 factorial design did not allow us to study the potential nonlinear effects of temperature and feeding frequency on life history traits, although such nonlinear effects have been shown in previous studies (Brett, 1971). Although this remains to be investigated in more detail, our results highlight the importance of considering the interactions between temperature, body size, and food to understand how larger predatory species respond to global changes in terms of developmental and life history strategies. In addition, the ecological consequences of temperature‐induced changes in body size are multiple. For instance, it can alter predator–prey size ratio which has important implications for the occurrence and strength of predator–prey interactions and thus for community dynamics and food web structure (Emmerson & Raffaelli, 2004; Kalinkat et al., 2013; Sentis et al., 2017; Vagnon et al., 2021; Williams & Martinez, 2000; Yodzis & Innes, 1992). To date, studies examining the consequences of temperature‐induced body size shifts on trophic interactions, community dynamics, and food web structure, only considered the reduction in adult size (Bideault et al., 2019; Osmond et al., 2017; Sentis et al., 2017). However, our results emphasize the importance of considering ontogeny in future studies as the temperature effect on growth is dependent on life stages. In addition, we expect phenological and geographic changes to alter the quantity and quality of resources (Ekvall et al., 2013; Paerl, 2014; Paerl & Huisman, 2008; Urrutia‐Cordero et al., 2017; Winder & Schindler, 2004), for example in predator–prey relationships by inducing temporal or spatial mismatches where the predator is left with reduced food (Boukal et al., 2019; Twining et al., 2022). These phenological asynchronies can alter the structure and dynamics of food webs and modify ecosystem processes (Damien & Tougeron, 2019; Renner & Zohner, 2018). Altogether, these studies indicate that it is important to investigate the direct effects of temperature as well as indirect effects such as altered food quality and availability to better understand the impact of climate change on growth, survival, and fecundity.

## AUTHOR CONTRIBUTIONS

**Simon Bazin:** Formal analysis (equal); writing – original draft (lead); writing – review and editing (lead). **Claire Hemmer‐Brepson:** Conceptualization (equal); data curation (equal); methodology (equal). **Maxime Logez:** Formal analysis (equal); writing – original draft (supporting); writing – review and editing (supporting). **Arnaud Sentis:** Formal analysis (equal); funding acquisition (equal); supervision (equal); validation (equal); writing – original draft (supporting); writing – review and editing (supporting). **Martin Daufresne:** Conceptualization (equal); funding acquisition (equal); methodology (equal); supervision (equal); validation (equal); writing – original draft (supporting); writing – review and editing (supporting).

## FUNDING INFORMATION

This work was supported by the ANR project EcoTeBo (ANR‐19‐CE02‐0001‐01) from the French National Research Agency (ANR) to A. S.

### OPEN RESEARCH BADGES

This article has earned Open Data and Open Materials badges. Data and materials are available at https://doi.org/10.6084/m9.figshare.20375850.v15.

## Supporting information


Appendix S1

## Data Availability

Data, scripts, and code are available online: https://doi.org/10.6084/m9.figshare.20375850.v15.

## References

[ece310770-bib-0001] Angilletta, M. J., Jr. , Steury, T. D. , & Sears, M. W. (2004). Temperature, growth rate, and body size in ectotherms: Fitting pieces of a life‐history puzzle. Integrative and Comparative Biology, 44(6), 498–509. 10.1093/icb/44.6.498 21676736

[ece310770-bib-0002] Arendt, J. D. (2011). Size‐fecundity relationships, growth trajectories, and the temperature‐size rule for ectotherms. Evolution, 65(1), 43–51. 10.1111/j.1558-5646.2010.01112.x 20812979

[ece310770-bib-0003] Arendt, J. (2007). Ecological correlates of body size in relation to cell size and cell number: Patterns in flies, fish, fruits and foliage. Biological Reviews, 82(2), 241–256. 10.1111/j.1469-185X.2007.00013.x 17437559

[ece310770-bib-0004] Atkinson, D. (1994). Temperature and organism size: A biological law for ectotherms? Advances in Ecological Research, 25, 1–58.

[ece310770-bib-0005] Atkinson, D. , & Sibly, R. M. (1997). Why are organisms usually bigger in colder environments? Making sense of a life history puzzle. Trends in Ecology & Evolution, 12(6), 235–239. 10.1016/S0169-5347(97)01058-6 21238056

[ece310770-bib-0006] Bates, D. , Mächler, M. , Bolker, B. , & Walker, S. (2015). Fitting linear mixed‐effects models using Lme4. Journal of Statistical Software, 67, 1–48. 10.18637/jss.v067.i01

[ece310770-bib-0007] Berrigan, D. , & Charnov, E. L. (1994). Reaction norms for age and size at maturity in response to temperature: A puzzle for life historians. Oikos, 70(3), 474–478. 10.2307/3545787

[ece310770-bib-0008] Bestion, E. , Teyssier, A. , Richard, M. , Clobert, J. , & Cote, J. (2015). Live fast, die young: Experimental evidence of population extinction risk due to climate change. PLoS Biology, 13(10), e1002281. 10.1371/journal.pbio.1002281 26501958 PMC4621050

[ece310770-bib-0009] Betini, G. S. , Wang, X. , Avgar, T. , Guzzo, M. M. , & Fryxell, J. M. (2020). Food availability modulates temperature‐dependent effects on growth, reproduction, and survival in *Daphnia magna* . Ecology and Evolution, 10(2), 756–762. 10.1002/ece3.5925 32015841 PMC6988562

[ece310770-bib-0010] Bideault, A. , Loreau, M. , & Gravel, D. (2019). Temperature modifies consumer‐resource interaction strength through its effects on biological rates and body mass. Frontiers in Ecology and Evolution, 7, 45. 10.3389/fevo.2019.00045

[ece310770-bib-0011] Boersma, M. , & Vijverberg, J. (1996). Food effects on life history traits and seasonal dynamics of Ceriodaphnia Pulchella. Freshwater Biology, 35(1), 25–34. 10.1046/j.1365-2427.1996.00478.x

[ece310770-bib-0012] Bogdan, A. , Levin, S. C. , Salguero‐Gómez, R. , & Knight, T. M. (2021). Demographic analysis of an Israeli Carpobrotus population. PLoS One, 16(4), e0250879. 10.1371/journal.pone.0250879 33930061 PMC8087044

[ece310770-bib-0013] Boggs, C. L. , & Ross, C. L. (1993). The effect of adult food limitation on life history traits in Speyeria Mormonia (Lepidoptera: Nymphalidae). Ecology, 74(2), 433–441. 10.2307/1939305

[ece310770-bib-0014] Boukal, D. S. , Bideault, A. , Carreira, B. M. , & Sentis, A. (2019). Species interactions under climate change: Connecting kinetic effects of temperature on individuals to community dynamics. Current Opinion in Insect Science, 35, 88–95. 10.1016/j.cois.2019.06.014 31445412

[ece310770-bib-0015] Brett, J. R. (1971). Energetic responses of Salmon to temperature. A study of some thermal relations in the physiology and freshwater ecology of sockeye Salmon (*Oncorhynchus nerkd*). American Zoologist, 11(1), 99–113. 10.1093/icb/11.1.99

[ece310770-bib-0016] Brett, J. R. , Shelbourn, J. E. , & Shoop, C. T. (1969). Growth rate and body composition of fingerling sockeye Salmon, Oncorhynchus Nerka, in relation to temperature and ration size. Journal of the Fisheries Research Board of Canada, 26(9), 2363–2394. 10.1139/f69-230

[ece310770-bib-0017] Brown, J. H. , Gillooly, J. F. , Allen, A. P. , Savage, V. M. , & West, G. B. (2004). Toward a metabolic theory of ecology. Ecology, 85(7), 1771–1789. 10.1890/03-9000

[ece310770-bib-0018] Clissold, F. J. , & Simpson, S. J. (2015). Temperature, food quality and life history traits of herbivorous insects. Current Opinion in Insect Science, 11, 63–70. 10.1016/j.cois.2015.10.011 28285760

[ece310770-bib-0019] Cole, L. C. (1954). The population consequences of life history phenomena. The Quarterly Review of Biology, 29(2), 103–137. 10.1086/400074 13177850

[ece310770-bib-0020] Corrêa, C. P. , Parreiras, S. S. , Beijo, L. A. , de Ávila, P. M. , Teixeira, I. R. V. , & Barchuk, A. R. (2021). Life history trait response to ambient temperature and food availability variations in the bean weevil Zabrotes Subfasciatus. Physiological Entomology, 46(3–4), 189–199. 10.1111/phen.12358

[ece310770-bib-0021] Courtney Jones, S. K. , Munn, A. J. , Penman, T. D. , & Byrne, P. G. (2015). Long‐term changes in food availability mediate the effects of temperature on growth, development and survival in striped marsh frog larvae: Implications for captive breeding programmes. Conservation Physiology, 3(1), cov029. 10.1093/conphys/cov029 27293714 PMC4778449

[ece310770-bib-0022] Cross, W. F. , Hood, J. M. , Benstead, J. P. , Huryn, A. D. , & Nelson, D. (2015). Interactions between temperature and nutrients across levels of ecological organization. Global Change Biology, 21(3), 1025–1040. 10.1111/gcb.12809 25400273

[ece310770-bib-0023] Damien, M. , & Tougeron, K. (2019). Prey–predator phenological mismatch under climate change. Current Opinion in Insect Science, 35, 60–68. 10.1016/j.cois.2019.07.002 31401300

[ece310770-bib-0024] Daufresne, M. , Lengfellner, K. , & Sommer, U. (2009). Global warming benefits the small in aquatic ecosystems. Proceedings of the National Academy of Sciences of the United States of America, 106(31), 12788–12793. 10.1073/pnas.0902080106 19620720 PMC2722360

[ece310770-bib-0025] Denny, J. , Spehar, R. , Mead, K. , & Yousuff, S. (1991). *Guidelines for culturing the Japanese Medaka*, *‘Oryzias Latipes’* . PB92137496. Environmental Research Lab.‐Duluth, MN.; AScI Corp., Duluth, MN. https://ntrl.ntis.gov/NTRL/dashboard/searchResults/titleDetail/PB92137496.xhtml

[ece310770-bib-0026] Dhillon, R. S. , & Fox, M. G. (2004). Growth‐independent effects of temperature on age and size at maturity in Japanese Medaka (*Oryzias latipes*). Copeia, 2004(1), 37–45. 10.1643/CI-02-098R1

[ece310770-bib-0027] Ding, L. , Kuhne, W. W. , Hinton, D. E. , Song, J. , & Dynan, W. S. (2010). Quantifiable biomarkers of normal aging in the Japanese Medaka fish (*Oryzias latipes*). PLoS One, 5(10), e13287. 10.1371/journal.pone.0013287 20949019 PMC2952620

[ece310770-bib-0028] Egami, N. , & Etoh, H. (1969). Life span data for the small fish, *Oryzias latipes* . Experimental Gerontology, 4(2), 127–129. 10.1016/0531-5565(69)90035-7 5353609

[ece310770-bib-0029] Ekvall, M. K. , de la Calle, J. , Martin, E. J. , Faassen, S. G. , Lürling, M. , & Hansson, L.‐A. (2013). Synergistic and species‐specific effects of climate change and water colour on cyanobacterial toxicity and bloom formation. Freshwater Biology, 58(11), 2414–2422. 10.1111/fwb.12220

[ece310770-bib-0030] Ellner, S. P. , Childs, D. Z. , & Rees, M. (2016). Data‐driven modelling of structured populations: A practical guide to the integral projection model (1st ed., 2016 edition). Springer.

[ece310770-bib-0031] Elser, J. J. , Bracken, M. E. S. , Cleland, E. E. , Gruner, D. S. , Stanley Harpole, W. , Hillebrand, H. , Ngai, J. T. , Seabloom, E. W. , Shurin, J. B. , & Smith, J. E. (2007). Global analysis of nitrogen and phosphorus limitation of primary producers in freshwater, marine and terrestrial ecosystems. Ecology Letters, 10(12), 1135–1142. 10.1111/j.1461-0248.2007.01113.x 17922835

[ece310770-bib-0032] Emmerson, M. C. , & Raffaelli, D. (2004). Predator–prey body size, interaction strength and the stability of a real food web. Journal of Animal Ecology, 73(3), 399–409. 10.1111/j.0021-8790.2004.00818.x

[ece310770-bib-0033] Enquist, B. J. , West, G. B. , Charnov, E. L. , & Brown, J. H. (1999). Allometric scaling of production and life‐history variation in vascular plants. Nature, 401(6756), 907–911.

[ece310770-bib-0034] Falkowski, P. G. , Barber, R. T. , & Smetacek, V. (1998). Biogeochemical controls and feedbacks on ocean primary production. Science, 281(5374), 200–206. 10.1126/science.281.5374.200 9660741

[ece310770-bib-0035] Forster, J. , Hirst, A. G. , & Atkinson, D. (2012). Warming‐induced reductions in body size are greater in aquatic than terrestrial species. Proceedings of the National Academy of Sciences of the United States of America, 109(47), 19310–19314. 10.1073/pnas.1210460109 23129645 PMC3511100

[ece310770-bib-0036] Frazier, M. R. , Arthur Woods, H. , & Harrison, J. F. (2001). Interactive effects of rearing temperature and oxygen on the development of *Drosophila melanogaster* . Physiological and Biochemical Zoology, 74(5), 641–650. 10.1086/322172 11517449

[ece310770-bib-0037] Fryxell, D. C. , Hoover, A. N. , Alvarez, D. A. , Arnesen, F. J. , Benavente, J. N. , Moffett, E. R. , Kinnison, M. T. , Simon, K. S. , & Palkovacs, E. P. (2020). Recent warming reduces the reproductive advantage of large size and contributes to evolutionary downsizing in nature. Proceedings. Biological Sciences, 287(1928), 20200608. 10.1098/rspb.2020.0608 32486974 PMC7341922

[ece310770-bib-0038] Gardner, J. L. , Peters, A. , Kearney, M. R. , Joseph, L. , & Heinsohn, R. (2011). Declining body size: A third universal response to warming? Trends in Ecology & Evolution, 26(6), 285–291. 10.1016/j.tree.2011.03.005 21470708

[ece310770-bib-0039] Giberson, D. J. , & Rosenberg, D. M. (1992). Effects of temperature, food quantity, and Nymphal rearing density on life‐history traits of a northern population of Hexagenia (Ephemeroptera:Ephemeridae). Journal of the North American Benthological Society, 11(2), 181–193. 10.2307/1467384

[ece310770-bib-0040] Giebelhausen, B. , & Lampert, W. (2001). Temperature reaction norms of *Daphnia magna*: The effect of food concentration. Freshwater Biology, 46(3), 281–289. 10.1046/j.1365-2427.2001.00630.x

[ece310770-bib-0041] Gray, E. M. (2013). Thermal acclimation in a complex life cycle: The effects of larval and adult thermal conditions on metabolic rate and heat resistance in Culex Pipiens (Diptera: Culicidae). Journal of Insect Physiology, 59(10), 1001–1007. 10.1016/j.jinsphys.2013.08.001 23932965

[ece310770-bib-0042] Gredilla, R. , Sanz, A. , Lopez‐Torres, M. , & Barja, G. (2001). Caloric restriction decreases mitochondrial free radical generation at complex I and lowers oxidative damage to mitochondrial DNA in the rat heart. The FASEB Journal, 15(9), 1589–1591. 10.1096/fj.00-0764fje 11427495

[ece310770-bib-0043] Hirshfield, M. F. (1980). An experimental analysis of reproductive effort and cost in the Japanese Medaka, *Oryzias latipes* . Ecology, 61(2), 282–292. 10.2307/1935187

[ece310770-bib-0044] Hoefnagel, K. N. , & Verberk, W. C. E. P. (2015). Is the temperature‐size rule mediated by oxygen in aquatic ectotherms? Journal of Thermal Biology, 54, 56–65. 10.1016/j.jtherbio.2014.12.003 26615727

[ece310770-bib-0045] Horne, C. R. , Hirst, A. G. , & Atkinson, D. (2015). Temperature‐size responses match latitudinal‐size clines in arthropods, revealing critical differences between aquatic and terrestrial species. Ecology Letters, 18(4), 327–335. 10.1111/ele.12413 25682961

[ece310770-bib-0046] Huey, R. B. , & Kingsolver, J. G. (2019). Climate warming, resource availability, and the metabolic meltdown of ectotherms. American Naturalist, 194, E140–E150. 10.1086/705679 31738103

[ece310770-bib-0047] Johnston, I. A. , Manthri, S. , Alderson, R. , Campbell, P. , Mitchell, D. , Whyte, D. , Dingwall, A. , Nickell, D. , Selkirk, C. , & Robertson, B. (2002). Effects of dietary protein level on muscle cellularity and flesh quality in Atlantic Salmon with particular reference to gaping. Aquaculture, 210(1), 259–283. 10.1016/S0044-8486(01)00862-6

[ece310770-bib-0048] Jones, O. R. , Barks, P. , Stott, I. , James, T. D. , Levin, S. , Petry, W. K. , Capdevila, P. , Che‐Castaldo, J. , Jackson, J. , Römer, G. , Schuette, C. , Thomas, C. C. , & Salguero‐Gómez, R. (2022). Rcompadre and rage—Two R packages to facilitate the use of the COMPADRE and COMADRE databases and calculation of life‐history traits from matrix population models. Methods in Ecology and Evolution, 13(4), 770–781. 10.1111/2041-210X.13792

[ece310770-bib-0049] Kalinkat, G. , Schneider, F. D. , Digel, C. , Guill, C. , Rall, B. C. , & Brose, U. (2013). Body masses, functional responses and predator–prey stability. Ecology Letters, 16(9), 1126–1134. 10.1111/ele.12147 23819684

[ece310770-bib-0050] Kiełbasa, A. , Walczyńska, A. , Fiałkowska, E. , Pajdak‐Stós, A. , & Kozłowski, J. (2014). Seasonal changes in the body size of two rotifer species living in activated sludge follow the temperature‐size rule. Ecology and Evolution, 4(24), 4678–4689. 10.1002/ece3.1292 25558362 PMC4278820

[ece310770-bib-0051] Kingsolver, J. G. , & Huey, R. B. (2008). Size, temperature, and fitness: Three rules. Evolutionary Ecology Research, 10, 251–268.

[ece310770-bib-0052] Kingsolver, J. G. , Shlichta, J. G. , Ragland, G. J. , & Massie, K. R. (2006). Thermal reaction norms for Caterpillar growth depend on diet. Evolutionary Ecology Research, 8(4), 703–715.

[ece310770-bib-0053] Kingsolver, J. G. , & Woods, H. A. (1998). Interactions of temperature and dietary protein concentration in growth and feeding of *Manduca sexta* caterpillars. Physiological Entomology, 23(4), 354–359. 10.1046/j.1365-3032.1998.234105.x

[ece310770-bib-0054] Koussoroplis, A.‐M. , & Wacker, A. (2016). Covariance modulates the effect of joint temperature and food variance on ectotherm life‐history traits. Ecology Letters, 19(2), 143–152. 10.1111/ele.12546 26612682

[ece310770-bib-0055] Lee, K. P. , & Roh, C. (2010). Temperature‐by‐nutrient interactions affecting growth rate in an insect ectotherm. Entomologia Experimentalis et Applicata, 136(2), 151–163. 10.1111/j.1570-7458.2010.01018.x

[ece310770-bib-0056] Lemoine, N. P. , & Burkepile, D. E. (2012). Temperature‐induced mismatches between consumption and metabolism reduce consumer fitness. Ecology, 93(11), 2483–2489. 10.1890/12-0375.1 23236919

[ece310770-bib-0057] Levin, S. C. , Childs, D. Z. , Compagnoni, A. , Evers, S. , Knight, T. M. , & Salguero‐Gómez, R. (2021). Ipmr: Flexible implementation of integral projection models in R. Methods in Ecology and Evolution, 12(10), 1826–1834. 10.1111/2041-210X.13683

[ece310770-bib-0058] Marn, N. , Jusup, M. , Legović, T. , Kooijman, S. A. L. M. , & Klanjšček, T. (2017). Environmental effects on growth, reproduction, and life‐history traits of loggerhead turtles. Ecological Modelling, 360, 163–178. 10.1016/j.ecolmodel.2017.07.001

[ece310770-bib-0059] McLeod, I. M. , Rummer, J. L. , Clark, T. D. , Jones, G. P. , McCormick, M. I. , Wenger, A. S. , & Munday, P. L. (2013). Climate change and the performance of larval coral reef fishes: The interaction between temperature and food availability. Conservation Physiology, 1(1), cot024. 10.1093/conphys/cot024 27293608 PMC4732438

[ece310770-bib-0060] Meyer, J. L. , Sale, M. J. , Mulholland, P. J. , & LeRoy Poff, N. (1999). Impacts of climate change on aquatic ecosystem functioning and health. Journal of the American Water Resources Association, 35(6), 1373–1386. 10.1111/j.1752-1688.1999.tb04222.x

[ece310770-bib-0061] Osmond, M. M. , Barbour, M. A. , Bernhardt, J. R. , Pennell, M. W. , Sunday, J. M. , & O'Connor, M. I. (2017). Warming‐induced changes to body size stabilize consumer‐resource dynamics. The American Naturalist, 189(6), 718–725. 10.1086/691387 28514639

[ece310770-bib-0062] Paerl, H. , & Huisman, J. (2008). Blooms like it hot. Science, 320, 57–58. 10.1126/science.1155398 18388279

[ece310770-bib-0063] Paerl, H. W. (2014). Mitigating harmful cyanobacterial blooms in a human‐ and climatically‐impacted world. Life, 4(4), 988–1012. 10.3390/life4040988 25517134 PMC4284478

[ece310770-bib-0064] Parmesan, C. , & Yohe, G. (2003). A globally coherent fingerprint of climate change impacts across natural systems. Nature, 421(6918), 37–42. 10.1038/nature01286 12511946

[ece310770-bib-0065] Perrin, N. (1995). About Berrigan and Charnov's life‐history puzzle. Oikos, 73(1), 137–139. 10.2307/3545737

[ece310770-bib-0066] Persson, J. , Wojewodzic, M. W. , Hessen, D. O. , & Andersen, T. (2011). Increased risk of phosphorus limitation at higher temperatures for *Daphnia magna* . Oecologia, 165(1), 123–129. 10.1007/s00442-010-1756-4 20803219 PMC3015186

[ece310770-bib-0067] Petersen, C. H. R. , Arthur Woods, H. , & Kingsolver, J. O. E. L. G. (2000). Stage‐specific effects of temperature and dietary protein on growth and survival of *Manduca sexta* caterpillars. Physiological Entomology, 25(1), 35–40. 10.1046/j.1365-3032.2000.00163.x

[ece310770-bib-0068] Pifferi, F. , Terrien, J. , Marchal, J. , Dal‐Pan, A. , Djelti, F. , Hardy, I. , Chahory, S. , Cordonnier, N. , Desquilbet, L. , Hurion, M. , Zahariev, A. , Chery, I. , Zizzari, P. , Perret, M. , Epelbaum, J. , Blanc, S. , Picq, J. L. , Dhenain, M. , & Aujard, F. (2018). Caloric restriction increases lifespan but affects brain integrity in Grey mouse lemur primates. Communications Biology, 1(1), 1–8. 10.1038/s42003-018-0024-8 30271916 PMC6123706

[ece310770-bib-0069] Pritchard, D. W. , Paterson, R. A. , Bovy, H. C. , & Barrios‐O'Neill, D. (2017). Frair: An R package for fitting and comparing consumer functional responses. Methods in Ecology and Evolution, 8(11), 1528–1534. 10.1111/2041-210X.12784

[ece310770-bib-0070] Rasmussen, R. S. , & Ostenfeld, T. H. (2000). Influence of growth rate on white muscle dynamics in rainbow trout and brook trout. Journal of Fish Biology, 56(6), 1548–1552. 10.1111/j.1095-8649.2000.tb02164.x

[ece310770-bib-0071] Renner, S. S. , & Zohner, C. M. (2018). Climate change and phenological mismatch in trophic interactions among plants, insects, and vertebrates. Annual Review of Ecology, Evolution, and Systematics, 49(1), 165–182. 10.1146/annurev-ecolsys-110617-062535

[ece310770-bib-0072] Rohner, P. T. , Blanckenhorn, W. U. , & Schäfer, M. A. (2017). Critical weight mediates sex‐specific body size plasticity and sexual dimorphism in the yellow dung fly *Scathophaga stercoraria* (Diptera: Scathophagidae). Evolution & Development, 19(3), 147–156. 10.1111/ede.12223 28463473

[ece310770-bib-0073] Sentis, A. , Binzer, A. , & Boukal, D. S. (2017). Temperature‐size responses Alter food chain persistence across environmental gradients. Ecology Letters, 20(7), 852–862. 10.1111/ele.12779 28544190

[ece310770-bib-0074] Sentis, A. , Haegeman, B. , & Montoya, J. M. (2022). Stoichiometric constraints modulate temperature and nutrient effects on biomass distribution and community stability. Oikos, 2022(7). 10.1111/oik.08601 PMC7614052 36644620

[ece310770-bib-0075] Sentis, A. , Morisson, J. , & Boukal, D. S. (2015). Thermal acclimation modulates the impacts of temperature and enrichment on trophic interaction strengths and population dynamics. Global Change Biology, 21(9), 3290–3298. 10.1111/gcb.12931 25808556

[ece310770-bib-0076] Sheridan, J. A. , & Bickford, D. (2011). Shrinking body size as an ecological response to climate change. Nature Climate Change, 1(8), 401–406. 10.1038/nclimate1259

[ece310770-bib-0077] Smallegange, I. M. , Caswell, H. , Toorians, M. E. M. , & de Roos, A. M. (2017). Mechanistic description of population dynamics using dynamic energy budget theory incorporated into integral projection models. Methods in Ecology and Evolution, 8(2), 146–154. 10.1111/2041-210X.12675

[ece310770-bib-0078] Sohal, R. S. , & Weindruch, R. (1996). Oxidative stress, caloric restriction, and aging. Science, 273(5271), 59–63. 10.1126/science.273.5271.59 8658196 PMC2987625

[ece310770-bib-0079] Sohlström, E. H. , Archer, L. C. , Gallo, B. , Jochum, M. , Kordas, R. L. , Rall, B. C. , Rosenbaum, B. , & O'Gorman, E. J. (2021). Thermal Acclimation Increases the Stability of a Predator–Prey Interaction in Warmer Environments. Global Change Biology, 27(16), 3765–3778. 10.1111/gcb.15715 34009702

[ece310770-bib-0080] Speakman, J. R. (2005). Body size, energy metabolism and lifespan. Journal of Experimental Biology, 208(9), 1717–1730. 10.1242/jeb.01556 15855403

[ece310770-bib-0081] Sterner, R. W. , & Elser, J. J. (2017). Ecological stoichiometry: The biology of elements from molecules to the biosphere. In Ecological stoichiometry. Princeton University Press.

[ece310770-bib-0082] Su, Y.‐S. , & Yajima, M. (2015). R2jags: Using R to Run ‘JAGS’ . R package version 0.5‐7 34.

[ece310770-bib-0083] Terao, O. (1985). Contribution to the study of the ecology of the medaka, Oryzias latipes, under natural conditions: Life span, reproduction, food habits and its seasonal changes. University of Tokyo.

[ece310770-bib-0084] Therneau, T. M. (2015). Coxme: Mixed effects cox models . R package version 2 (3).

[ece310770-bib-0085] Therneau, T. M. , & Lumley, T. (2015). Package ‘Survival’. *R Top Doc* 128 (10), 28–33.

[ece310770-bib-0086] Thierry, B. (2005). Integrating proximate and ultimate causation: Just one more go! Current Science, 89(7), 1180–1183.

[ece310770-bib-0087] Thomas, M. K. , Aranguren‐Gassis, M. , Kremer, C. T. , Gould, M. R. , Anderson, K. , Klausmeier, C. A. , & Litchman, E. (2017). Temperature–nutrient interactions exacerbate sensitivity to warming in phytoplankton. Global Change Biology, 23(8), 3269–3280. 10.1111/gcb.13641 28132424

[ece310770-bib-0088] Thunell, V. , Gårdmark, A. , Huss, M. , & Vindenes, Y. (2023). Optimal energy allocation trade‐off driven by size‐dependent physiological and demographic responses to warming. Ecology, 104(4), e3967. 10.1002/ecy.3967 36565169

[ece310770-bib-0089] Tilman, D. (1982). Resource competition and community structure. Princeton University Press. 7162524

[ece310770-bib-0090] Twining, C. W. , Ryan Shipley, J. , & Matthews, B. (2022). Climate change creates nutritional phenological mismatches. Trends in Ecology & Evolution, 37, 736–739. 10.1016/j.tree.2022.06.009 35811171

[ece310770-bib-0091] Urrutia‐Cordero, P. , Ekvall, M. K. , Ratcovich, J. , Soares, M. , Wilken, S. , Zhang, H. , & Hansson, L.‐A. (2017). Phytoplankton diversity loss along a gradient of future warming and brownification in freshwater mesocosms. Freshwater Biology, 62(11), 1869–1878. 10.1111/fwb.13027

[ece310770-bib-0092] Uszko, W. , Huss, M. , & Gårdmark, A. (2022). Smaller species but larger stages: Warming effects on inter‐ and intraspecific community size structure. Ecology, 103, e3699. 10.1002/ecy.3699 35352827 PMC9285768

[ece310770-bib-0093] Vagnon, C. , Cattanéo, F. , Goulon, C. , Grimardias, D. , Guillard, J. , & Frossard, V. (2021). An allometric niche model for species interactions in temperate freshwater ecosystems. Ecosphere, 12(3), e03420. 10.1002/ecs2.3420

[ece310770-bib-0094] Verberk, W. C. E. P. , David Atkinson, K. , Hoefnagel, N. , Hirst, A. G. , Horne, C. R. , & Siepel, H. (2021). Shrinking body sizes in response to warming: Explanations for the temperature–size rule with special emphasis on the role of oxygen. Biological Reviews, 96(1), 247–268. 10.1111/brv.12653 32959989 PMC7821163

[ece310770-bib-0095] Visser, M. E. , & Both, C. (2005). Shifts in phenology due to global climate change: The need for a yardstick. Proceedings of the Royal Society B: Biological Sciences, 272(1581), 2561–2569. 10.1098/rspb.2005.3356 PMC1559974 16321776

[ece310770-bib-0096] Von Bertalanffy, L. U. D. W. I. G. (1938). A quantitative theory of organic growth (inquiries on growth laws. II). Human Biology, 10(2), 181–213.

[ece310770-bib-0097] Walczyńska, A. , Kiełbasa, A. , & Sobczyk, M. (2016). ‘Optimal thermal range’ in ectotherms: Defining criteria for tests of the temperature‐size‐rule. Journal of Thermal Biology, 60, 41–48. 10.1016/j.jtherbio.2016.06.006 27503715

[ece310770-bib-0098] Walczyńska, A. , Labecka, A. M. , Sobczyk, M. , Czarnoleski, M. , & Kozłowski, J. (2015). The temperature–size rule in *Lecane inermis* (Rotifera) is adaptive and driven by nuclei size adjustment to temperature and oxygen combinations. Journal of Thermal Biology, 54, 78–85. 10.1016/j.jtherbio.2014.11.002 26615729

[ece310770-bib-0099] Walczyńska, A. , & Sobczyk, Ł. (2017). The underestimated role of temperature–oxygen relationship in large‐scale studies on size‐to‐temperature response. Ecology and Evolution, 7(18), 7434–7441. 10.1002/ece3.3263 28944028 PMC5606864

[ece310770-bib-0100] Walters, R. J. , & Hassall, M. (2006). The temperature‐size rule in ectotherms: May a general explanation exist after all? The American Naturalist, 167(4), 510–523. 10.1086/501029 16670994

[ece310770-bib-0101] Williams, R. J. , & Martinez, N. D. (2000). Simple rules yield complex food webs. Nature, 404(6774), 180–183. 10.1038/35004572 10724169

[ece310770-bib-0102] Winder, M. , & Schindler, D. E. (2004). Climate change uncouples trophic interactions in an aquatic ecosystem. Ecology, 85(8), 2100–2106. 10.1890/04-0151

[ece310770-bib-0103] Wojewodzic, M. W. , Kyle, M. , Elser, J. J. , Hessen, D. O. , & Andersen, T. (2011). Joint effect of phosphorus limitation and temperature on alkaline phosphatase activity and somatic growth in *Daphnia magna* . Oecologia, 165(4), 837–846. 10.1007/s00442-010-1863-2 21153741 PMC3056991

[ece310770-bib-0104] Yamamoto, T. (1975). Medaka (Killifish). Biology and Strains, 365.

[ece310770-bib-0105] Yodzis, P. , & Innes, S. (1992). Body size and consumer‐resource dynamics. American Naturalist, 139(6), 1151–1175. 10.1086/285380

[ece310770-bib-0106] Zamudio, K. R. , Huey, R. B. , & Crill, W. D. (1995). Bigger isn't always better: Body size, developmental and parental temperature and male territorial success in *Drosophila melanogaster* . Animal Behaviour, 49(3), 671–677. 10.1016/0003-3472(95)80200-2

[ece310770-bib-0107] Zuo, W. , Moses, M. E. , West, G. B. , Hou, C. , & Brown, J. H. (2012). A general model for effects of temperature on ectotherm ontogenetic growth and development. Proceedings of the Royal Society B: Biological Sciences, 279(1734), 1840–1846. 10.1098/rspb.2011.2000 PMC3297449 22130604

