# Supplementary material for: Distinct impacts of feeding frequency and warming on life history traits affect population fitness in vertebrate ectotherms
Source: Ecol Evol. 2023 Nov 23;13(11):e10770. doi: 10.1002/ece3.10770 (PMC10667609; doi:10.1002/ece3.10770)
Supplement: Supplementary file 1 — Appendix S1 [file ECE3-13-e10770-s001.docx]

# **Supporting information**


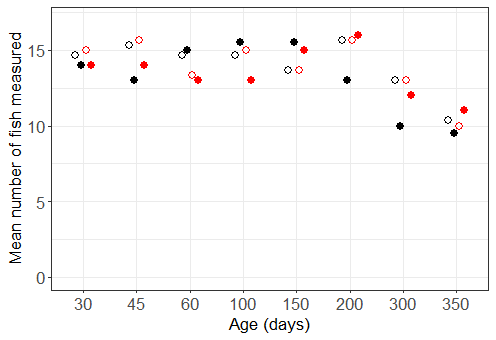


**Fig. S 1:** Mean number of fish measured at different ages. Jittered black and red points correspond to the mean number of fish measured in the cold and warm treatments, respectively. Filled and empty circles correspond to the continuous and intermittent feeding treatments, respectively.


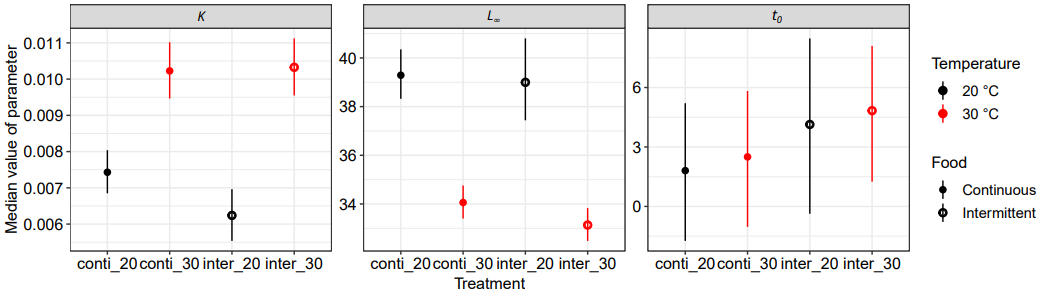


**Fig. S 2 :** Estimated Von Bertallanfy parameters for each treatment. Black and red colors correspond to the cold and warm treatments, respectively. Filled and empty circles correspond to the median of continuous and intermittent feeding treatments, respectively. Bars represent 95 % credibility intervals.

Consistent with the experimental curves and TSR, warming significantly increased the initial growth rate *K* and decreased the maximum asymptotic size *L_∞_*. Intermittent feeding had no effect on the initial growth rate *K* for fish reared at 30 °C, but significantly reduced *K* for fish reared at 20 °C. At the end of our experiment, the adult size of intermittent-fed fish was smaller than that of continuously-fed fish, especially at 20 °C. Yet, intermittent feeding had no significant effect on the maximum asymptotic size *L_∞_*, indicating that beyond 350 days, fish should reach the same size regardless of their food condition. Extrapolating to 700 days (life span of a medaka) from our experimental curves, intermittent-fed fish should reach the same adult size as continuously-fed fish at approximately 400 and 300 days under cold and warm conditions, respectively (Fig. S 4). The theoretical age at which body size is zero *t_0_* was not significantly different between temperature conditions.


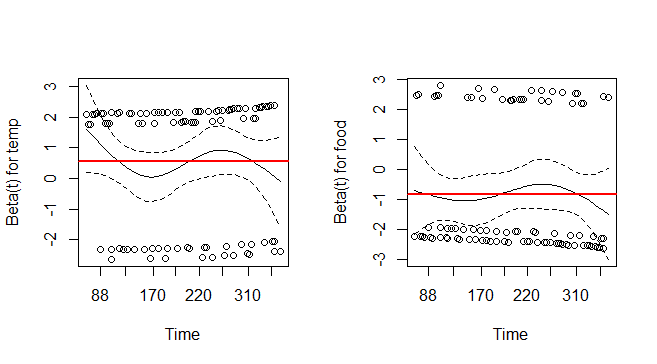


**Fig. S 3:** Cox model assumption of hazard proportionality for temperature and food condition.

The *cox.zph* function correlates the scaled Schoenfeld residuals with time for each covariate to test for independence between residuals and time. Additionally, it performs a global test for the model as a whole. From our model output, this test was not statistically significant for temperature
(*chi² = 0.20, df = 1, p = 0.65*), feeding frequency (*chi² = 0.00, df = 1, p = 0.97*) and the global test
(*chi² = 0.20, df = 2, p = 0.90*), indicating a proportional hazards.


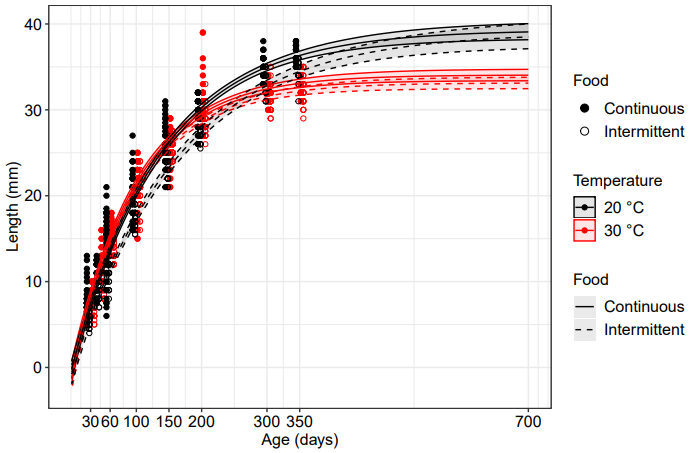


**Fig. S 4:** Extrapolation of Von Bertalanffy growth curve for each combination of temperature and food conditions. Black and red colors represent the cold and warm treatments, respectively. Solid and dotted lines represent the continuous and intermittent feeding treatments, respectively. Areas represent the 95 % credibility intervals. Points represent experimentally measured sizes at age.

**Table S 1:** Responses in size at maturity and adult size to warming and food increase in the experimental studies. Symbols +, - and x indicate a positive, negative or no effect of the variable, respectively.

| Reference | Taxa | Variable | Size at maturity | Adult size | Somatic Growth Rate |
| --- | --- | --- | --- | --- | --- |
| (Betini et al. 2020) | Daphnia *Daphnia magna* | Warming | x | - |  |
|  |  | Food | x | + |  |
| (Brett, Shelbourn, and Shoop 1969) | Fish Stockeye Salmon  *Oncorhynchus nerka* | Warming |  |  |  |
|  |  | Food |  |  |  |
|  |  | Interaction |  |  | Optimum growth temperature  shifts to the left as the food is reduced |
| (Courtney Jones et al. 2015) | Frog *Limnodynastes peronii* | Warming | - |  |  |
|  |  | Food | - |  |  |
| (Giberson and Rosenberg 1992) | Ephemeridae *Hexagenia limbata Hexagenia rigida* | Warming |  | + |  |
|  |  | Food |  | + |  |
| (Giebelhausen and Lampert 2001) | Daphnia *Daphnia magna* | Warming | - |  |  |
|  |  | Food | - |  |  |
| (Kiełbasa et al. 2014) | Rotifers *Lecane inermis* *Cephalodella gracilis* | Warming |  |  |  |
|  |  | Food |  |  |  |
|  |  | Interaction |  | Adult size reduction with only 1 of 2  nutritional qualities |  |
| (Kingsolver and Woods 1998) | Caterpillar *Manduca sexta* | Warming |  |  | + |
|  |  | Food |  |  | x or - |
|  |  | Interaction |  |  | x |
| (Lee and Roh 2010) | Caterpillar *Spodoptera exigua* | Warming |  |  |  |
|  |  | Food |  |  |  |
|  |  | Interaction | Mass at pupation reduction under extreme food conditions |  |  |
| (Marn et al. 2017) | Loggerhead turtles *Caretta caretta* | Warming | x | x |  |
|  |  | Food | x | + |  |
| (McLeod et al. 2013) | Anemonefish *Amphiprion percula* | Warming | x |  |  |
|  |  | Food | x |  |  |
| (Persson et al. 2011) | Daphnia *Daphnia magna* | Warming |  |  | + |
|  |  | Food |  |  | - |
|  |  | Interaction |  |  | The higher the temperature, the more phosphorus limitation decreased the SGR |
| (Petersen, Woods, and Kingsolver 2000) | Caterpillar *Manduca sexta* | Warming |  |  | + |
|  |  | Food |  |  | x |
|  |  | Interaction |  |  | x |
| (Rohner, Blanckenhorn, and Schäfer 2017) | Yellow dung fly *Scathophaga stercoraria* | Warming | x |  |  |
|  |  | Food | - |  |  |
| (Wojewodzic et al. 2011) | Daphnia *Daphnia magna* | Warming |  |  | + |
|  |  | Food |  |  | - |
|  |  | Interaction |  |  | The higher the temperature, the more phosphorus limitation decreased the SGR |


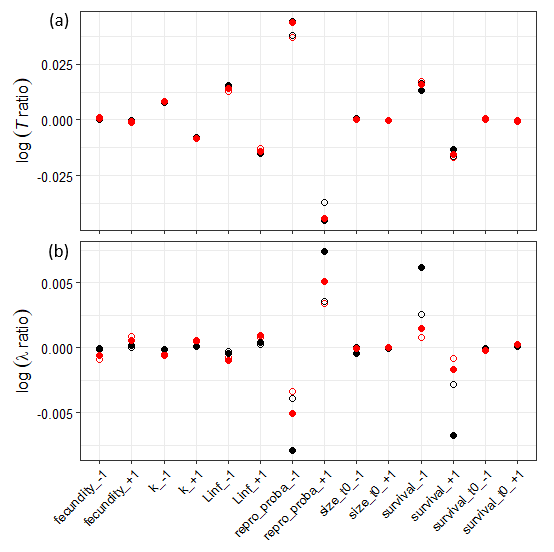


**Fig. S 5**: Sensitivity analysis of (a) generation time *T* and (b) asymptotic per capita population growth rate *λ*. Black and red colors correspond to the cold and warm treatments, respectively. Filled and empty circles correspond to the median of continuous and intermittent feeding treatments, respectively.

We carried out sensitivity analyses to investigate the sensitivity of generation time *T* and the asymptotic per capita population growth rate *λ* to small changes in the values of vital rates. To do so, we added or subtracted 1 % to the slope of the relationships between survival, reproductive probability or fecundity and body size. For the parameters that are independent of body size, we added or subtracted 1 % to the mean value. For the growth function, we also modified *K* and *L_∞_* by adding or subtracting 1 % to their mean values. We then investigated the sensitivity of *T* and *λ* by calculating the log ratio of the parameter (*λ* or *T*) estimated by the model with a change of 1 % in a single variable to the parameter estimated by the baseline IPM model. The further the log of this ratio is away from 0, the more sensitive the demographic parameter is to the vital rate.

Demographic parameters are most sensitive to variability in reproductive probability (Fig. S 5). Since we consider all fish to reproduce with probability = 1 from sexual maturity, adding or substracting 1 % to the slope of the regression is equivalent to increasing or decreasing age at sexual maturity by 13.7, 10.3, 2.4, and 1.8 days for conditions conti_20, inter_20, conti_30, and inter_30, respectively. Not surprisingly, this input strongly influences the demographic parameters since in the model the length of time a fish is fertile depends directly on age at maturity. Demographic parameters are also sensitive to the probability of survival. As with the probability of reproduction, this survival probability also determines the length of time a fish can produce eggs before it dies. Finally, the generation time is somewhat sensitive to the *K* and *L_∞_* parameters of the Von Bertallanfy model. By influencing growth, these parameters will determine the rate at which a fish reaches size at sexual maturity in the model, and thus the rate at which a fish can reproduce, directly affecting generation time.

# **References**

Betini, Gustavo S., Xueqi Wang, Tal Avgar, Matthew M. Guzzo, and John M. Fryxell. 2020. ‘Food Availability Modulates Temperature-Dependent Effects on Growth, Reproduction, and Survival in Daphnia Magna’. *Ecology and Evolution* 10 (2): 756–62. https://doi.org/10.1002/ece3.5925.

Brett, JR, JE Shelbourn, and CT Shoop. 1969. ‘Growth Rate and Body Composition of Fingerling Sockeye Salmon, Oncorhynchus Nerka, in Relation to Temperature and Ration Size’. *Journal of the Fisheries Research Board of Canada* 26 (9): 2363–94. https://doi.org/10.1139/f69-230.

Courtney Jones, Stephanie K., Adam J. Munn, Trent D. Penman, and Phillip G. Byrne. 2015. ‘Long-Term Changes in Food Availability Mediate the Effects of Temperature on Growth, Development and Survival in Striped Marsh Frog Larvae: Implications for Captive Breeding Programmes’. *Conservation Physiology* 3 (1): cov029. https://doi.org/10.1093/conphys/cov029.

Giberson, Donna J., and David M. Rosenberg. 1992. ‘Effects of Temperature, Food Quantity, and Nymphal Rearing Density on Life-History Traits of a Northern Population of Hexagenia (Ephemeroptera:Ephemeridae)’. *Journal of the North American Benthological Society* 11 (2): 181–93. https://doi.org/10.2307/1467384.

Giebelhausen, B., and W. Lampert. 2001. ‘Temperature Reaction Norms of Daphnia Magna: The Effect of Food Concentration’. *Freshwater Biology* 46 (3): 281–89. https://doi.org/10.1046/j.1365-2427.2001.00630.x.

Kiełbasa, Anna, Aleksandra Walczyńska, Edyta Fiałkowska, Agnieszka Pajdak-Stós, and Jan Kozłowski. 2014. ‘Seasonal Changes in the Body Size of Two Rotifer Species Living in Activated Sludge Follow the Temperature-Size Rule’. *Ecology and Evolution* 4 (24): 4678–89. https://doi.org/10.1002/ece3.1292.

Kingsolver, J. G., and H. A. Woods. 1998. ‘Interactions of Temperature and Dietary Protein Concentration in Growth and Feeding of Manduca Sexta Caterpillars’. *Physiological Entomology* 23 (4): 354–59. https://doi.org/10.1046/j.1365-3032.1998.234105.x.

Lee, Kwang Pum, and Chris Roh. 2010. ‘Temperature-by-Nutrient Interactions Affecting Growth Rate in an Insect Ectotherm’. *Entomologia Experimentalis et Applicata* 136 (2): 151–63. https://doi.org/10.1111/j.1570-7458.2010.01018.x.

Marn, Nina, Marko Jusup, Tarzan Legović, S. A. L. M. Kooijman, and Tin Klanjšček. 2017. ‘Environmental Effects on Growth, Reproduction, and Life-History Traits of Loggerhead Turtles’. *Ecological Modelling* 360 (September): 163–78. https://doi.org/10.1016/j.ecolmodel.2017.07.001.

McLeod, Ian M., Jodie L. Rummer, Timothy D. Clark, Geoffrey P. Jones, Mark I. McCormick, Amelia S. Wenger, and Philip L. Munday. 2013. ‘Climate Change and the Performance of Larval Coral Reef Fishes: The Interaction between Temperature and Food Availability’. *Conservation Physiology* 1 (1). https://doi.org/10.1093/conphys/cot024.

Persson, Jonas, Marcin Włodzimierz Wojewodzic, Dag Olav Hessen, and Tom Andersen. 2011. ‘Increased Risk of Phosphorus Limitation at Higher Temperatures for Daphnia Magna’. *Oecologia* 165 (1): 123–29. https://doi.org/10.1007/s00442-010-1756-4.

Petersen, C.H.ristine, H. Arthur Woods, and J.o.e.l. G. Kingsolver. 2000. ‘Stage-Specific Effects of Temperature and Dietary Protein on Growth and Survival of Manduca Sexta Caterpillars’. *Physiological Entomology* 25 (1): 35–40. https://doi.org/10.1046/j.1365-3032.2000.00163.x.

Rohner, Patrick T., Wolf U. Blanckenhorn, and Martin A. Schäfer. 2017. ‘Critical Weight Mediates Sex-Specific Body Size Plasticity and Sexual Dimorphism in the Yellow Dung Fly Scathophaga Stercoraria (Diptera: Scathophagidae)’. *Evolution & Development* 19 (3): 147–56. https://doi.org/10.1111/ede.12223.

Wojewodzic, Marcin W., Marcia Kyle, James J. Elser, Dag O. Hessen, and Tom Andersen. 2011. ‘Joint Effect of Phosphorus Limitation and Temperature on Alkaline Phosphatase Activity and Somatic Growth in Daphnia Magna’. *Oecologia* 165 (4): 837–46. https://doi.org/10.1007/s00442-010-1863-2.
